# P-504. Disparities in PrEP Coverage by Sex Among Individuals Diagnosed with Bacterial STIs in a Large Municipal Healthcare System

**DOI:** 10.1093/ofid/ofae631.703

**Published:** 2025-01-29

**Authors:** Emma Kaplan-Lewis, Eunice Casey, Kruti Gala, Zeyu Li, Xingyu Dai, Gabriel Cohen

**Affiliations:** NYC Health and Hospitals, NY, New York; NYC Health and Hospitals, NY, New York; Health + Hospitals, New York, New York; NYC Health + Hospitals, New York, New York; NYC Health + Hospitals, New York, New York; NYC Health + Hospitals, New York, New York

## Abstract

**Background:**

Nationally almost 20% of new HIV diagnosis are in females. Females carry 53% of sexually transmitted infection (STI) disease burden in the US, yet nationally represent only 10% of individuals on Pre-Exposure Prophylaxis (PrEP). The US Preventive Services Task Force and CDC include females diagnosed with bacterial STIs as one of the populations to be considered for PrEP. However, females are often not the focus of HIV PrEP campaigns, resulting in knowledge gaps, bias and stigma that further challenge PrEP access for women. As part of efforts to improve sexual health services and access within NYC's municipal healthcare system, we designed a sexual health dashboard (SHD) that included a “PrEP Eligible” metric focused on federal PrEP guidelines related to bacterial STIs.

**Figure:**
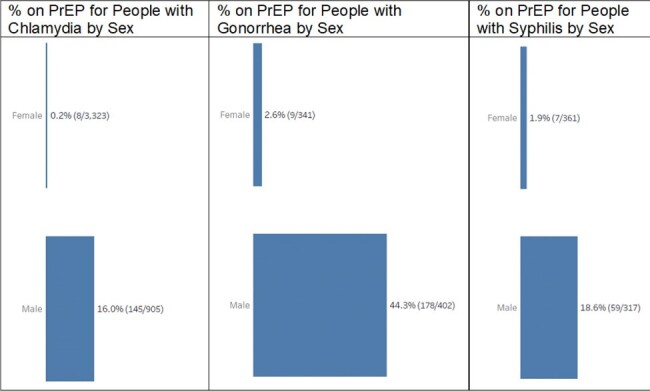

**Methods:**

All alive patients aged 13-75 years old with a primary care or women’s health clinic visit within a NYC Health and Hospitals facility during April 2023-March 2024 were included in the SHD. The SHD displays testing and positivity rates for Chlamydia, Gonorrhea, and Syphilis, in addition to PrEP eligibility and PrEP active status. PrEP eligibility is based on high frequency of HIV or STI tests (3+ in 1 year), an abnormal STI lab or STI diagnosis and ‘PrEP Active’ is defined as an active prescription for PrEP within the preceding 6-months.

**Results:**

Females represented 82% of PrEP eligible patients but only 13.2% (162) of PrEP Active patients (1,223). Among all people with Chlamydia, 0.2% of females were PrEP Active compared to 16% of males. For Gonorrhea, 2.6% of females were PrEP Active compared to 44.3% of males. For Syphilis, 1.9% of females were PrEP Active compared to 18.6% of males (Figure 1).

**Conclusion:**

According to the CDC, 6% of sexually acquired HIV can be attributed to bacterial STIs, however PrEP usage remains low among eligible females within a municipal healthcare system in NYC. Dedicated efforts are being made within NYC Health and Hospitals to expand PrEP to primary care and women’s health, with the hopes that diversifying care settings for PrEP will reach populations not currently accessing PrEP services. Lack of recognition of the connection between HIV risk and STIs may disproportionately impact females and contribute to underutilization of PrEP.

**Disclosures:**

**Emma Kaplan-Lewis, MD**, Gilead Pharmaceuticals: Grant/Research Support

